# Protection of *Salvia miltiorrhizae* to the Spleen and Thymus of Rats with Severe Acute Pancreatitis or Obstructive Jaundice

**DOI:** 10.1155/2009/186136

**Published:** 2009-11-16

**Authors:** Zhang Xiping, Li Chuyang, Zhang Jie, Ruan Yuefang, Ma Meili

**Affiliations:** ^1^Department of General Surgery, Hangzhou First People's Hospital, Hangzhou 310006, Zhejiang Province, China; ^2^Library, Hangzhou First People's Hospital, Hangzhou 310006, Zhejiang Province, China; ^3^The First College of Clinical Medicine, Zhejiang University of Traditional Chinese Medicine, Hangzhou 3100053, China; ^4^Department of Nephropathy, 117 Hospital of People's Liberation Army, Hangzhou 310013, Zhejiang Province, China

## Abstract

*Objective*. To investigate the therapeutic effects and mechanism of *Salvia miltiorrhizae* in the treatment of SAP and OJ. *Methods*. A total of 288 rats were used for SAP- and OJ-associated experiments. The rats were randomly divided into sham-operated group, model control group and treated group. The mortality rates of rats, contents of endotoxin and PLA_2_ in blood, patholodgical changes of different indexes in spleen and thymus were observed. *Results*. The contents of endotoxin and PLA2 in treated group were significantly lower than those in model control group.The pathological severity scores of spleen and thymus of SAP rats as well as that of spleen of OJ rats in treated groups were significantly lower than those in model control groups (*P* < .05). The staining intensity as well as the product of the staining intensity and positive rate of Bax protein of spleen in model control group were significantly higher than those in treated groups (*P* < .01) , and the apoptosis index of spleen in treated group was significantly lower than that in model control group (*P* < .01). *Conclusion*. *Salvia miltiorrhizae* exerts protective effects on the spleen and thymus of SAP rats and spleen of OJ rats.

## 1. Introduction

Since severe acute pancreatitis (SAP) and obstructive jaundice (OJ) are common diseases, the study of their pathogenesis and treatment has been a hot spot in medical field for a long time [1–4]. Severe acute pancreatitis (SAP) is a systemic disease resulted from pancreatic self-digestion and characterized by pancreatic necrosis. It can induce vascular leakage, shock, systemic inflammatory response syndrome (SIRS), and even multiple organ dysfunction (MODS) [5, 6]. Although SAP is dangerous, has many complications, and shows a higher mortality rate, the mechanism underlying this disease has not yet been fully clarified [7–9]. As the study of SIRS and MODS is deepened, the immune function injury in SAP has gradually attracted more attention. Some studies have shown that both pancreatic and extrapancreatic manifestations of SAP are closely related with immune function [10]. Also, the observation that low immune function is an important contributing factor for the death of OJ patients should not be ignored. Therefore, one of the most important approaches for treating SAP and OJ is to improve the function of the patient's immune system [11–14]. At present, the drugs that show specific effects on injuries of immune organs in SAP or OJ are lacking, and few studies have been found to investigate this kind of drugs. The development and utilization of traditional Chinese medicine show good prospects in the therapy of SAP and OJ since it has advantages of lower cost, more extensive pharmacological effects, and less side effects [15–18]. 

*Salvia miltiorrhizae* injection belongs to a traditional Chinese medicine injection that can improve microcirculation, scavenge oxygen free radicals, regulate the metabolism of inflammatory lipid mediators, protect intestinal mucosa, and decrease the levels of inflammatory mediators [19, 20]. Some scholars believe that *Salvia miltiorrhizae* also can enhance macrophage function, inhibit platelet aggregation, reduce blood viscosity, prevent microvascular coagulation, and relieve vascular spasm and occlusion [21]. Some clinical studies have shown that *Salvia miltiorrhizae*, as an auxiliary drug, have some therapeutic effects on SAP and OJ. *Salvia miltiorrhizae* is able to improve the clinical symptoms and significantly shorten the recovery duration of hematuria amylase levels and liver function damage, the remission duration of abdominal pain, as well as the duration of hospitalization in patients with SAP [22]. Moreover, *Salvia miltiorrhizae* can also protect intestinal mucosa, reduce the translocation of bacteria and endotoxins, enhance immunity, improve operative safety, and shorten healing duration in patients with OJ [23]. 

In the present study, we have applied tissue microarray technology to examine the pathological changes in the spleen and thymus of SAP and OJ rats, and investigated the protective effect of *Salvia miltiorrhizae* on the spleen and thymus of SAP and OJ rats and the associated mechanism. We hope this research can provide some valued materials for clinical application of *Salvia miltiorrhizae*.

## 2. Materials and Methods

### 2.1. Materials

A total of 288 healthy male SD rats of clean grade, weighing between 270 and 330 g, were provided by the Laboratory Animal Research Center of the Zhejiang University of Traditional Chinese Medicine (China); Sodium taurocholate and sodium pentobarbital were purchased from Sigma Corporation, USA; *Salvia miltiorrhizae* injection (also called Danshen injection, each 10 mL vial contains active components equivalent to 15 g of the original medicine) was purchased from Chiatai Qingchunbao Pharmaceutical Co., Ltd (China); Rabbit anti-mouse Bax antibody was purchased from Santa Cruz Biotechnology, Inc. (USA); TUNEL assay kit was purchased from Takara Bio Inc. (Japan). Endotoxin ELISA Kit was purchased from Associates of Cape Cord (USA), the calculation unit for content is EU/mL; The serum secretory phospholipase A_2_ enzyme Assay ELISA kit (PLA_2_) was purchased from R&D system Inc. (USA), the calculation unit for content is U/mL.

### 2.2. Methods

#### 2.2.1. Animal Grouping

A total of 108 rats were used for SAP-associated experiments and randomly divided into sham-operated group, model control group, and treated group (*n* = 36), which were further randomly subdivided into 3, 6, and 12 hours groups (*n* = 12) according to time points after operation. Another 180 rats were utilized for OJ-associated experiments and randomly divided into sham-operated group, model control group, and treated group (*n* = 60), which were further randomly subdivided into 7, 14, 21, and 28 d groups (*n* = 15) according to time duration after operation. SAP was an acute experiment. When the observering procedure time exceed 12 hours, the mortality rate will increase largely, resulting in sample numbers decrease largely and disadvantage to statistics. According to references exterior and interior of our contury, the 3, 6, 12 hour point was the most reasonable. However, OJ was a chronic experiment, according to refereences exterior and interior of our contury, the 7-, 14-, 21-, 28-day point was the most reasonable. 

#### 2.2.2. Preparation of SAP Models and Associated Therapeutic Regimens

The rats were anesthetized with an intraperitoneal injection of 2.5% sodium pentobarbital (0.2 mL/100 g). Under aseptic conditions, the thigh skin was cut open to expose femoral vein and a transfusion passage was established through which continuous infusion was maintained using a microinfusion pump (1 mL/h/100 g). Subsequently, a median abdominal wall incision was made to expose duodenal papilla, and a number 5 syringe needle was used to prick a small hole in the mesenteric avascular area. The epidural catheter was first inserted into duodenal cavity via the hole, and then placed into the bile-pancreatic duct toward the direction of papilla. The catheter head was temporarily clamped using a microvascular clamp, and another microvascular clamp was used to occlude the common bile duct at the confluence of hepatic ducts to prevent a backflow of injected drugs into the liver. After connecting the epidural catheter end with the transfusion converter, 3.5% sodium taurocholate (0.1 mL/100 g) was transfused at a flow rate of 0.2 mL/min using a microinjection pump (produced by Zhejiang University, China). After completing the transfusion, microvascular forceps and epidural catheter were maintained for further 4 minutes and then removed. A check-up was then conducted to see whether bile leakage was present. After suturing the hole in the lateral wall of the duodenum, the abdominal cavity was closed conventionally. Sham-operated groups were performed just by moving the pancreas and duodenum after opening the abdominal cavity. Fifteen minutes after successful operation, a single dose of *Salvia miltiorrhizae* injection (0.4 mL/100 g body weight [24–27]) was given via femoral vein to rats in treated groups while equal volume of physiological saline solution was used in the sham-operated and the model control group. Continuous infusion of physiological saline solution using a microinjection pump was then maintained until the end of the 3-, 6-, and 12-hour observation period in the corresponding groups.

#### 2.2.3. Preparation of OJ Models and Associated Therapeutic Regimens

After rats were anesthetized with an intraperitoneal injection of 2.5% sodium pentobarbital (0.2 mL/100 g), the abdominal cavity was opened to identify and dissociate common bile duct along the hepatoduodenal ligament. For rats in model control groups and the treated groups, the proximal end of common bile duct was double-ligated with surgical threads, common bile duct was cut off, and a layered suture of the abdominal wall was performed to close the abdominal cavity. For rats in sham-operated groups, common bile duct was only dissociated but not ligated, and a layered suture of the abdominal wall was also performed to close the abdominal cavity. An intraperitoneal injection of *Salvia miltiorrhizae* injection at a dose of 0.2 mL/100 g/d [24–27] was given to rats in treated groups while equal volume of physiological saline solution was used in the sham-operated groups and the model control groups. Injection was maintained until the end of the 7, 14, 21, and 28-day observation period in the corresponding groups.

### 2.3. Detecting Indexes

(1) At various time points after operation, the mortality rate of rats was recorded. At the corresponding time points after operation, SAP or OJ rats were anesthetized with 2.5% sodium pentobarbital and killed to take blood samples and tissue specimens of spleen and thymus that were subsequently subjected to pathological examination and morphological observation under a light microscope. At 12 hours after operation in SAP experiment, the ultrastructural changes in the spleen and thymus were also observed under an electron microscope. The pathological severity score of spleen and thymus in SAP groups and spleen in OJ groups were analyzed, respectively. 

(2) Determination of the contents of plasma endotoxin and serum PLA_2_, the determination of blood parameters was conducted according to the instructions provided with the kits. 

(3) Tissue microarray technology was used to prepare pathological sections of spleen and thymus, and then conducted immunohistochemical staining of Bax protein and TUNEL staining. The changes in the expression levels of Bax and the apoptosis index of lymphocyte in the spleen and thymus were observed. Firstly, tissue microarrays sections of spleen and thymus were prepared, the diameter was 1.5 mm. The Envision two-step method was used to detect the expression levels of Bax, a protein in the spleen and thymus. The evaluation standard were as follows: (1) the staining intensity was evaluated according to the extent of cell coloration: “ − ” represents negative staining; “+” represents mild staining, positively stained cells showed a yellow pigment; “++” represents moderate staining, positively stained cells showed a brown pigment; “+++” represents intense staining, positively stained cells showed a dark brown pigment, each of which was scored as 0, 1, 2, and 3 points, respectively, during statistical analysis; (2) the evaluation standard of the positive rate: none of positive cells (−); the percentage of positive cells was less than 25% (+); the percentage of positive cells ranged between 26% and 50% (++); the percentage of positive cells was more than 50% (+++), each of which was scored as 0, 1, 2, and 3 points, respectively, during statistical analysis. Then, DNA nick in situ end-labeling (TUNEL) staining in tissue microarrays sections of spleen and thymus were conducted according to the instructions provided with the kits. The apoptotic index was calculated. Apoptotic index= apoptotic cell count/total cell count × 100%. 

(4) The results were subjected to statistical analysis.

### 2.4. Statistical Analysis

The compiled data were first input into an Excel sheet, and then read into SPSS15.0 for further analysis. Normal data were expressed as means (standard deviation) while nonnormal data were expressed as medians (interquartile range). Analysis of variance and pairwise comparisons were used for normal data, whereas nonnormal data were subjected to nonparametric test, among which Kruskal-Wallis H test was used for pairwise comparisons and Mann-Whitney U test for multiple comparisons. Yates' chi-square test (*χ* ^2^) was used for intergroup comparisons of mortality rates.

## 3. Results

### 3.1. SAP-Associated Experiments

#### 3.1.1. Comparison of Mortality Rate

One and five rats died in model control groups at 3 and 12 hours after operation, respectively; three died in treated group at 12 hours after operation; no rats died in the remaining groups. There was no marked difference in mortality rate between the time points at 3 and 6 hours after operation. At 12 hours after operation, only the mortality rate in model control group was significantly higher than that in sham-operated group (*P* = .037), and no marked difference in mortality rate was observed among other groups (*P* > .05) [24]. 

#### 3.1.2. Comparison of the Content of Endotoxin in Plasma

At all time points after operation, the contents of plasma endotoxin in model control group were significantly higher than those in sham-operated group (*P* < .001). At 3 and 12 hours after operation, the contents in treated group were significantly higher than those in sham-operated group (*P* < .01). At 6 and 12 hours after operation, the contents in treated group were significantly lower than those in model control group (*P* < .01), see Table 1.

#### 3.1.3. Comparison of the Content of PLA_2_ in Serum

At all time points after operation, the contents of serum PLA_2_ in model control group and treated group were significantly higher than those in sham-operated group (*P* < .001). At 6 and 12 hours after operation, the contents in treated group were significantly lower than those in model control group (*P* < .01), see Table 1.

#### 3.1.4. Gross Pathological Changes and Pathological Changes under Light and Electron Microscopy of Spleen

**(1) Sham-Operated Group**

- *Gross Pathological Changes.* At all time points after operation, the morphology and structure of spleen were normal in each group.
- *Pathological Changes under Light Microscopy.* No marked difference in pathological changes was observed among each time point after operation. The spleen was normal in the majority of rats. The expansion of the blood sinuses in the red pulp of spleen as well as focal necrosis or spotty necrosis of the follicular germinal centers were seen in few rats.
- *Pathological Changes under Electron Microscopy.* The structure of lymphocytes was normal, and a great number of red blood cells were present in the spleen tissue.

**(2) Model Control Group**

**Figure 1:**
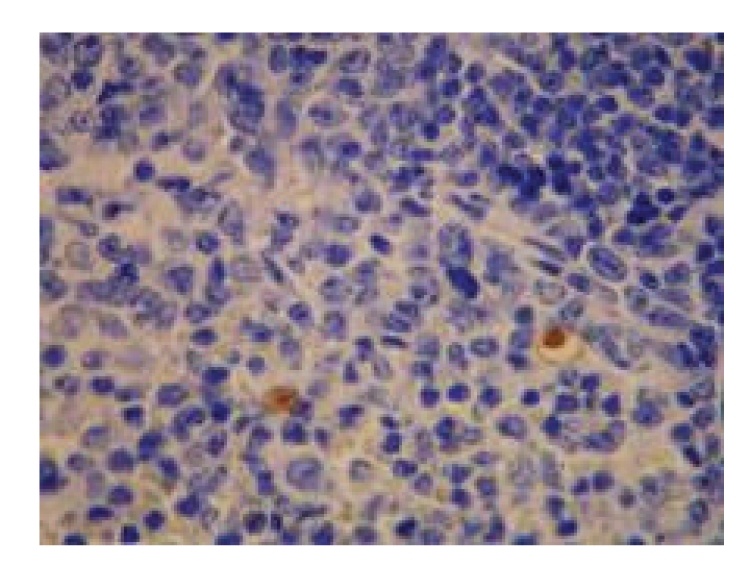
Model control group—6 hours in SAP experiment spleen (several apoptoic cells), TUNEL × 400.

- *Gross Pathological Changes. *At 3 hours after operation, spleen congestion was seen in few rats. At 6 and 12 hours after operation, spleen congestion was aggravated and the number of rats showing spleen congestion increased; purple black plaques were seen in some areas of spleen in some rats.
- *Pathological Changes under Light Microscopy. *No marked difference in pathological changes was observed among each time point after operation. The necrosis of the follicular centers and the expansion of the blood sinuses in the red pulp of spleen, some of which were accompanied by splenic arteriolar sclerosis were seen, see Figure 1.
- *Pathological Changes under Electron Microscopy. *The congestion of spleen and the apoptosis of lymphocytes were seen.

**(3) Treated Group**

**Figure 2:**
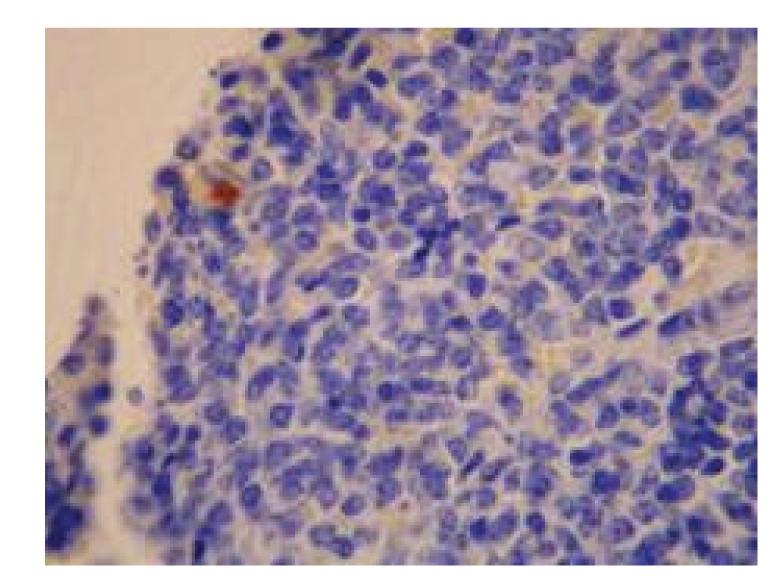
Treated group—6 hours in SAP experiment spleen (several apoptoic cells) TUNEL, × 400.

- *Gross Pathological Changes. *The congestion of spleen was mitigated. The number of rats showing spleen congestion and purple black plaques decreased.
- *Pathological Changes under Light Microscopy. *The pathological changes were slightly mitigated with the increase in therapeutic duration. At 3 hours after operation, the structure of the white pulp and red pulp was clear in the majority of rats; the necrosis of the follicular germinal centers and the expansion of the blood sinuses in the red pulp of spleen, some of which were accompanied by splenic arteriolar sclerosis, were seen in few rats. At 6 and 12 hours after operation, the structure of the white pulp and red pulp was clear and showed no abnormality in the majority of rats; the necrosis of the follicular centers and the expansion of the blood sinuses in the red pulp of spleen were seen in very few rats. see Figure 2.

**(4) Pathological Changes under Electron Microscopy**

Spleen congestion and mitochondrial vacuolation were seen.

#### 3.1.5. Comparison of the Pathological Severity Scores of Spleen

Because no severity score standard for pathological changes of spleen were reported in literature, we established the standard according to the extent of pathological damage of spleen, which were shown in Table 2. At all time points after operation, the pathological severity scores of spleen in model control group were significantly higher than those in sham-operated group (*P* < .05). At all time points after operation, no marked difference was noted between treated group and sham-operated group (*P* > .05). At 6 and 12 hours after operation, the scores in treated group were significantly lower than those in model control group (*P* < .05), see Table 3.

#### 3.1.6. Examination of Pathological Changes of Spleen

**(1) Comparison of the Staining Intensity of Bax Protein of Spleen**

At 12 hours after operation, the staining intensity of Bax protein of spleen in model control group was significantly higher than that in sham-operated group (*P* < .01). At all time points after operation, no significant difference was noted between sham-operated group and treated group as well as between model control group and treated group (*P* > .05), see Table 4.

**(2) Comparison of the Product of the Staining Intensity and Positive Rate of Bax Protein of Spleen**

At 12 hours after operation, the product of the staining intensity and positive rate of Bax protein of spleen in model control group was significantly higher than that in sham-operated group (*P* < .01). At 3 hours after operation, the product in treated group was significantly higher than that in sham-operated group (*P* < .01). At all time points after operation, no significant difference was noted between treated group and model control group (*P* > .05), see Table 4.

**(3) The Expression of NF-*κ*B Protein of Spleen**

The expression of NF-*κ*B protein of spleen was negative.

**(4) Comparison of the Apoptosis Index of Spleen**

At all time points after operation, no significant difference in the apoptosis index of spleen was noted between sham-operated group and model control group, between sham-operated group and treated group, as well as between model control group and treated group (*P* > .05), see Table 4.

#### 3.1.7. Gross Pathological Changes and Pathological Changes under Light or Electron Microscopy of Thymus

**(1) Sham-Operated Group**

**Figure 3:**
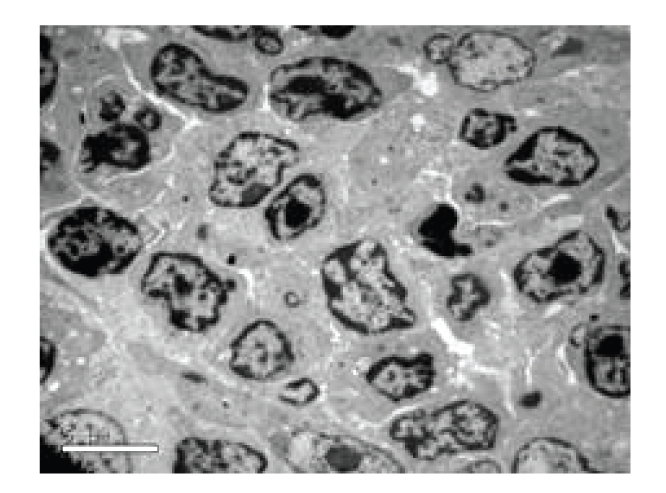
Sham-operated group—12 hours in SAP experiment thymus (normal), Electron microscope × 2550.

- *Gross Pathological Changes. *The morphology of thymus was normal.
- *Pathological Changes under Light Microscopy. *No significant difference in pathological changes was noted among each time point after operation. The thymic tissue was roughly normal in the majority of rats. Obscure boundary between thymic cortex and medulla was occasionally seen.
- *Pathological Changes under Electron Microscopy. *The structure of thymus was normal, see Figure 3.

**(2) Model Control Group**

**Figure 4:**
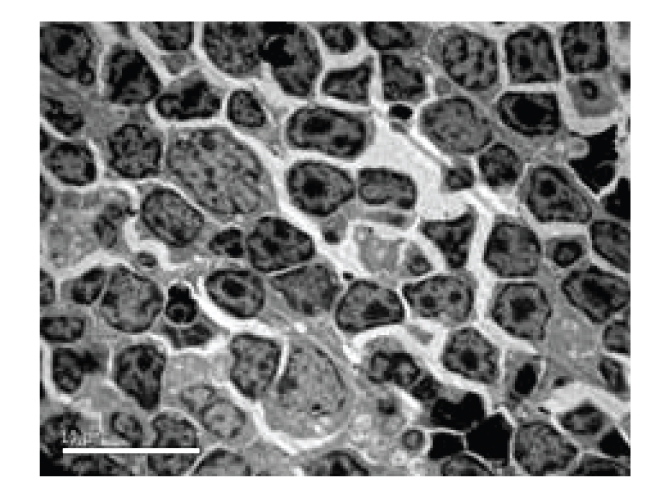
Model control group—12 hours in SAP experiment thymus (apoptoic cells obviously increase in thymus), Electron microscope × 1850.

- *Gross Pathological Changes. *The morphology of thymus was roughly normal.
- *Pathological Changes under Light Microscopy*. No significant difference in pathological changes was noted among each time point after operation. The thymic tissue was roughly normal in the majority of rats. Obscure boundary between thymic cortex and medulla was occasionally seen. Hemorrhage was seen in cortico-medullary junction region.
- *Pathological Changes under Electron Microscopy. *Thymic cells shrank. The relative distance between cells was widened. Many apoptotic cells were seen, see Figure 4.

**(3) Treated Group**

**Figure 5:**
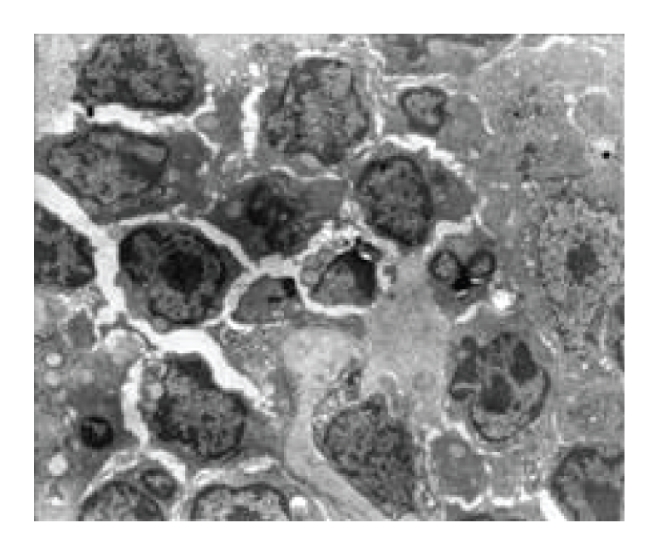
Treated group—12 hours in SAP experiment thymus (normal thymic lymphocyte and reticulate epithelium) Electron microscope × 5000.

- *Gross Pathological Changes. *The morphology of thymus was roughly normal.
- *Pathological Changes under Light Microscopy. *No significant difference in pathological changes was noted among each time point after operation. The thymic tissue was roughly normal in the majority of rats. Obscure boundary between thymic cortex and medulla was occasionally seen.
- *Pathological Changes under Electron Microscopy. *The structure of thymus was roughly normal, see Figure 5.

#### 3.1.8. Comparison of the Pathological Severity Scores of Thymus

The severity score of pathological changes of thymus was conducted according to the standard reported in literature [28]. At all time points after operation, no significant difference in pathological severity scores was noted between sham-operated group and model control group as well as between sham-operated group and treated group (*P* > .05). At 3 and 6 hours after operation, the pathological severity scores in treated group were significantly lower than those in model control group (*P* < .05), see Table 3.

#### 3.1.9. The Expression of Bax and NF-*κ*B Proteins of Thymus

The expression of Bax and NF-*κ*B proteins of spleen was negative.

### 3.2. OJ-Associated Experiments

#### 3.2.1. Comparison of Mortality Rate

It was noted that 2, 4, 4, and 7 rats died in model control groups on 7, 14, 21, and 28 days after operation, respectively; and 3, 2, and 4 rats died in treated groups on 14, 21, and 28 days after operation. The mortality rates on 7 days after operation showed no marked difference among three experimental groups. On 14 and 21 days after operation, the mortality rates in sham-operated groups were significantly lower than those in model control groups (*P* = .032). On 28 d after operation, the mortality rate in sham-operated group was lower than those in both the model control group (*P* = .006) and the treated group (*P* = .032), and the difference was statistically significant.

#### 3.2.2. Comparison of the Content of Endotoxin in Plasma

At all time points after operation, the contents of plasma endotoxin in sham-operated group were significantly lower than those in model control group and the treated group (*P* < .01), see Table 5.

#### 3.2.3. Comparison of the Content of PLA_2_ in Serum

On 14, 21, and 28 days after operation, the contents of serum PLA_2_ in sham-operated group were significantly lower than those in model control group and treated group (*P* < .01). On 28 days after operation, the content in treated group was significantly lower than that in model control group (*P* < .01), see Table 5.

#### 3.2.4. Gross Pathological Changes and Pathological Changes under Light Microscopy of Spleen

**(1) Sham-Operated Group**

- *Gross Pathological Changes. *The morphology of spleen was normal, and no obvious pathological changes were seen.
- *Pathological Changes under Light Microscopy. *The spleen was roughly normal in all rats.

**(2) Model Control Group**

**Figure 6:**
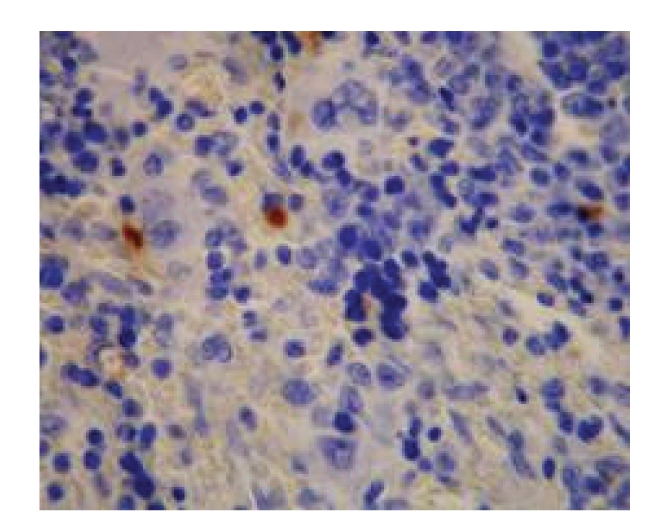
Model control group—28 days in OJ experiment spleen (several apoptoic cells), TUNEL × 400.

- *Gross Pathological Changes. *On 7 days after operation, the size of spleen increased by 1.2–1.5 times, the texture of spleen became fragile, and the color of spleen showed no change. On 14 days after operation, the spleen became enormous, with a thickness of above 0.6 cm in most parts; the color of spleen became deeper, and the texture of spleen became fragile. On 21 and 28 days after operation, the spleen became enormous, with an average size of 4 × 1*  * × 1 cm; the texture of spleen became fragile, and the color of spleen became purple black.
- *Pathological Changes under Light Microscopy. *On 7 days after operation, the spleen was roughly normal in all rats. On 14 days after operation, the fusion, enlargement, or spotty necrosis of follicular germinal centers in the white pulp of spleen, the hyperplasia of the fibrous tissue in the sinus, as well as splenic arteriolar sclerosis were seen in the majority of rats; the spleen was roughly normal in few rats. On 21 and 28 days after operation, the fusion, enlargement, or spotty necrosis of follicular germinal centers in the white pulp of spleen, the hyperplasia of the fibrous tissue in the sinus, as well as splenic arteriolar sclerosis were seen in few rats; the spleen was roughly normal in some rats, see Figure 6.

**(3) Treated Group**

**Figure 7:**
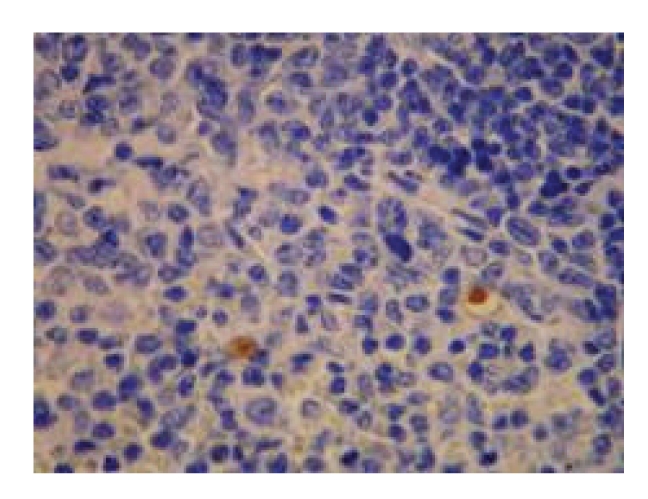
Treated group—28 days in OJ experiment spleen (several apoptoic cells), TUNEL × 400.

- *Gross Pathological Changes.* On 7 and 14 days after operation, no marked difference was observed when compared with those in model control group. On 21 and 28 days after operation, the size of spleen increased, with an average size of 3 × 1 × 0.5 cm.
- *Pathological Changes under Light Microscopy. *No significant difference in pathological changes was noted among each time point after operation. The spleen was roughly normal in the majority of rats. Splenic arteriolar sclerosis was seen in few rats, see Figure 7.

#### 3.2.5. Comparison of the Pathological Severity Scores of Spleen

 The severity score standard for pathological changes of spleen was cited in Table 2. On 21 days after operation, the pathological severity score of spleen in model control group was significantly higher than that in sham-operated group (*P* < .05). At all time points after operation, no marked difference was noted between treated group and sham-operated group (*P* > .05). On 21 and 28 days after operation, the pathological severity scores in treated group were significantly lower than those in model control group (*P* < .05), see Table 6.

#### 3.2.6. Examination of Pathological Changes of Spleen

**(1) Comparison of the Staining Intensity of Bax Protein of Spleen**

**Figure 8:**
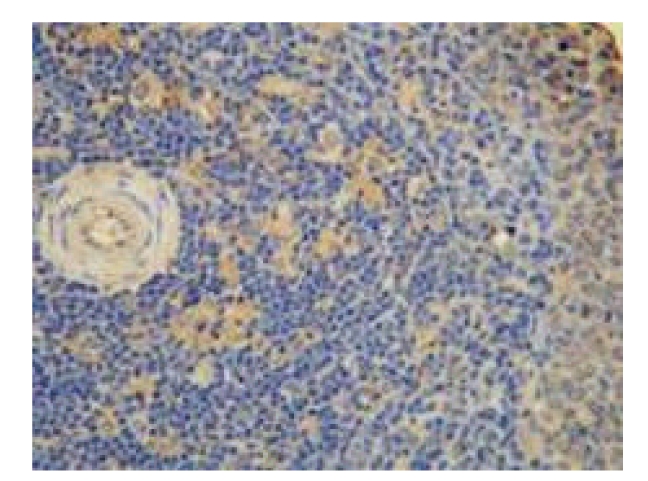
Model control group—21 d in OJ experiment (++) spleen, Bax × 200.

**Figure 9:**
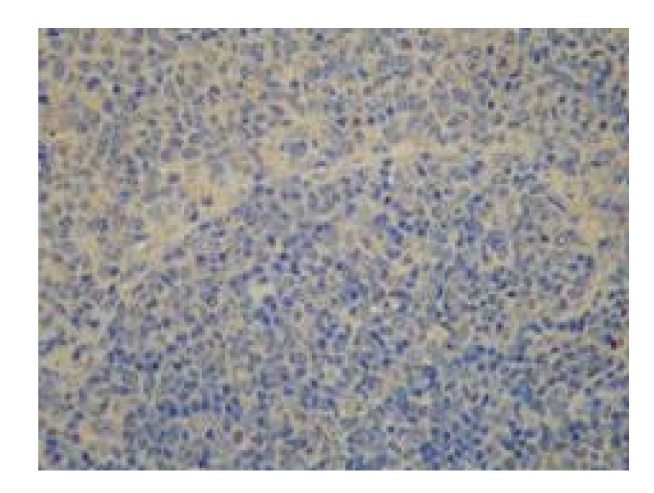
Treated group—21 d in OJ experiment (+) spleen, Bax × 200.

On 7, 14, and 21 days after operation, the staining intensity of Bax protein of spleen in model control group was significantly higher than that in sham-operated group (*P* < .01). On 7 days after operation, the staining intensity in treated group was significantly higher than that in sham-operated group (*P* < .01). On 21 days after operation, the staining intensity in model control group was significantly higher than that in treated group (*P* < .01), see Table 7 and Figures 8 and 9.

**(2) Comparison of the Product of the Staining Intensity and Positive Rate of Bax Protein of Spleen**

On 7, 14, and 21 days after operation, the products of the staining intensity and the positive rate of Bax protein of spleen in model control group were significantly higher than those in sham-operated group (*P* < .01). On 7 days after operation, the product in treated group was significantly higher than that in sham-operated group (*P* < .01). On 21 days after operation, the product in model control group was significantly higher than that in treated group (*P* < .01), see Table 7.

**(3) Comparison of the Staining Intensity of NF-*κ*B of Spleen**

At all time points after operation, no significant difference in the staining intensity of NF-*κ*B of spleen was noted between sham-operated group and model control group, between sham-operated group and treated group, as well as between model control group and treated group (*P* > .05), see Table 7.

**(4) Comparison of the Product of the Staining Intensity and the Positive Rate of NF-*κ*B Protein of Spleen**

At all time points after operation, no significant difference in the product of the staining intensity and the positive rate of NF-*κ*B protein of spleen was noted between sham-operated group and model control group, between sham-operated group and treated group, as well as between model control group and treated group (*P* > .05), see Table 7.

**(5) Comparison of the Apoptosis Index of Spleen**

On 7, 14, and 28 days after operation, the apoptosis indexes of spleen in model control group were significantly higher than those in sham-operated group (*P* < .01). At all time points after operation, no significant difference was noted between treated group and sham-operated group (*P* > .05). On 28 days after operation, the apoptosis index in treated group was significantly lower than that in model control group (*P* < .01), see Table 7.

#### 3.2.7. Gross Pathological Changes and Pathological Changes under Light Microscopy of Thymus

**(1) Sham-Operated Group**

**Figure 10:**
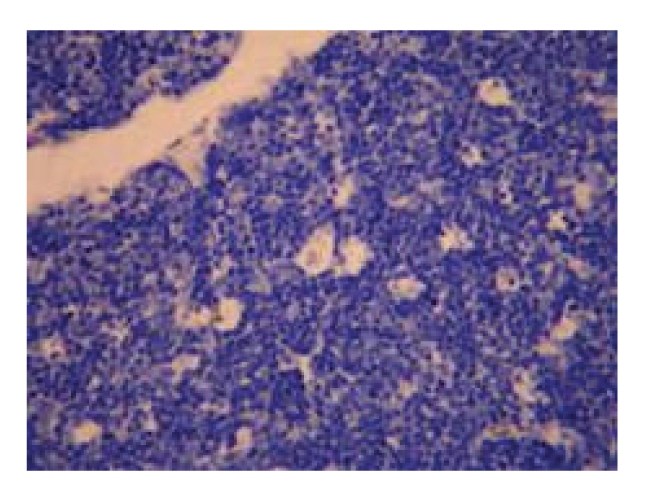
Sham-operated group—28 d in OJ experiment thymus (negative TUNEL × 200).

- *Pathological Changes under Light Microscopy.* No significant difference in pathological changes was noted among each time point after operation. The thymic tissue was roughly normal in all rats, see Figure 10.

**(2) Model Control Group**

**Figure 11:**
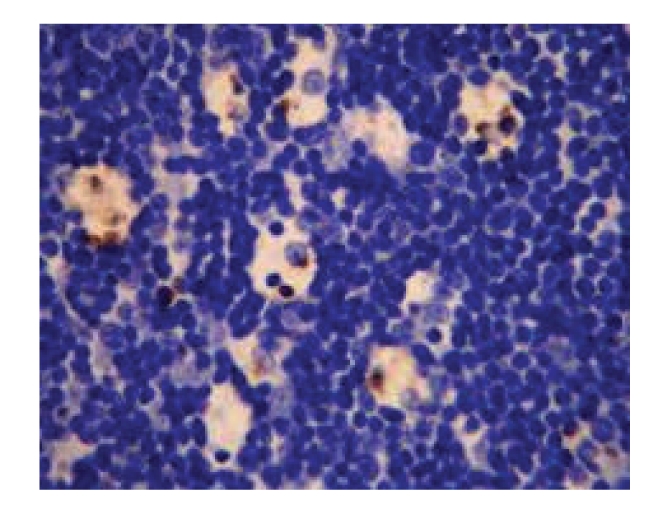
Model control group—28 d in OJ experiment thymus (many apoptoic cells), TUNEL × 400.

- *Gross Pathological Changes. *On 7 days after operation, the thymus slightly shrank in the majority of rats. On 14 days after operation, the thymus shrank and became jaundice in the majority of rats. On 21 and 28 days after operation, the thymus significantly shrank and became jaundice in all rats.
- *Pathological Changes under Light Microscopy*. No significant difference in pathological changes was noted among each time point after operation. The thymic tissue was roughly normal in the majority of rats. Obscure boundary between thymic cortex and medulla was occasionally seen. On 14 days after operation, the thymic tissue was roughly normal. The pathological changes were similar among 14, 21, and 28 days after operation: the thymic tissue was roughly normal in the majority of rats, and obscure boundary between thymic cortex and medulla was occasionally seen, see Figure 11.

**(3) Treated Group**

**Figure 12:**
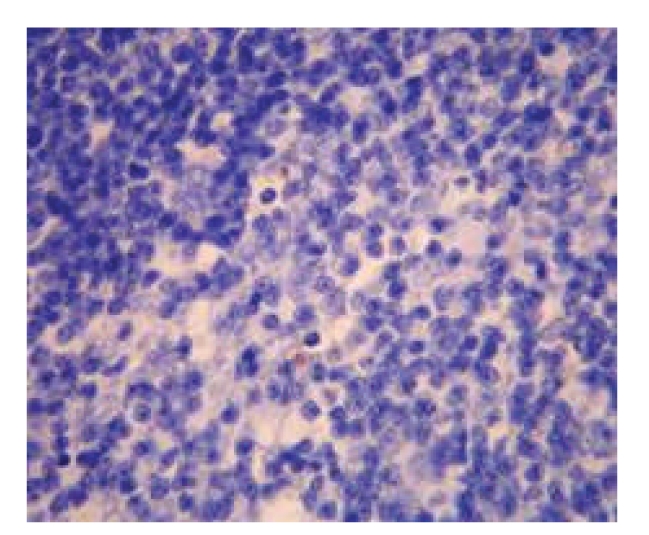
Treated group—28 d in OJ experiment thymus (several apoptoic cells), TUNEL × 400.

- *Gross Pathological Changes. *On 7 and 14 days after operation, no marked difference was observed when compared with those in model control group. On 21 and 28 days after operation, the thymus became slightly jaundice but showed no obvious shrinkage.
- *Pathological Changes under Light Microscopy. *No significant difference in pathological changes was noted among each time point after operation. The thymic tissue was normal. Obscure boundary between thymic cortex and medulla was occasionally seen. On 7, 21, and 28 days after operation, the thymic tissue was roughly normal in the majority of rats, and obscure boundary between thymic cortex and medulla of was seen in some rats. On 14 days after operation, the thymic tissue was roughly normal, see Figure 12.

#### 3.2.8. Examination of Pathological Changes of Thymus

**(1) Comparison of the Staining Intensity of Bax Protein of Thymus**

**Figure 13:**
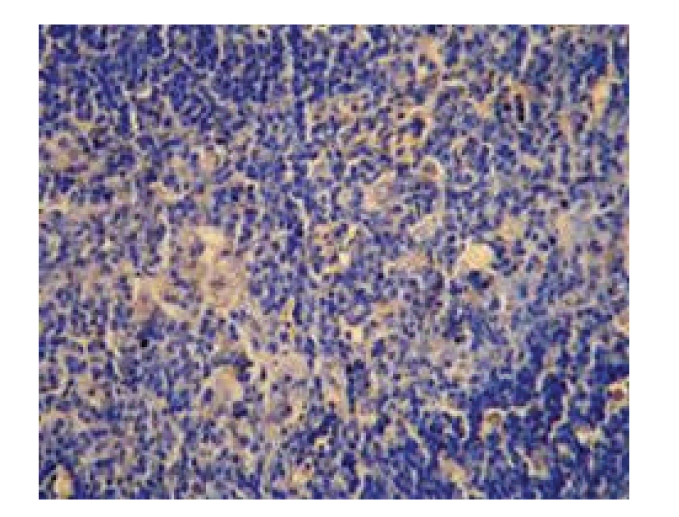
Model control group—7 d in OJ experiment thymus (+++), Bax × 200.

**Figure 14:**
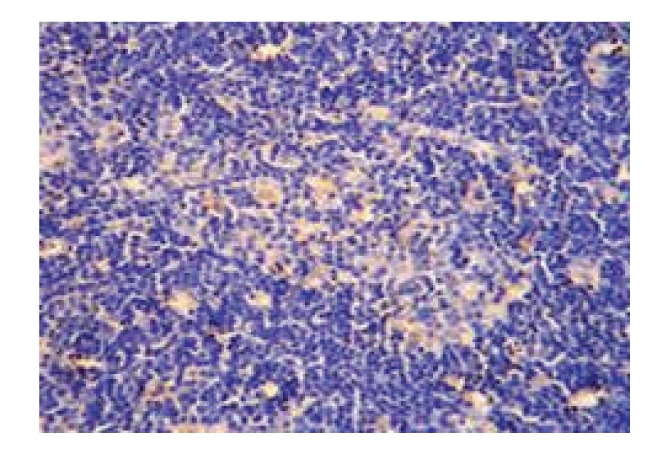
Model control group—21 d in OJ experiment thymus (++), Bax × 200.

On 7, 21, and 28 days after operation, the staining intensity of Bax protein of thymus in model control group was significantly higher than that in sham-operated group (*P* < .01). At all time points after operation, no significant difference was noted between treated group and sham-operated group as well as between treated group and model control group (*P* > .05), see Table 8 and Figures 13 and 14.

**(2) Comparison of the Product of the Staining Intensity and the Positive Rate of Bax Protein of Thymus**

On 7, 21, and 28 days after operation, the products of the staining intensity and the positive rate of Bax protein of thymus in model control group were significantly higher than those in sham-operated group (*P* < .01). At all time points after operation, no significant difference was noted between treated group and sham-operated group as well as between treated group and model control group (*P* > .05), see Table 8.

**(3) Comparison of the Staining Intensity of NF-*κ*B of Thymus**

At all time points after operation, no significant difference in the staining intensity of NF-*κ*B of thymus was noted between sham-operated group and model control group, between sham-operated group and treated group, as well as between model control group and treated group (*P* > .05). See Table 8.

**(4) Comparison of the Product of the Staining Intensity and the Positive Rate of NF-*κ*B Protein of Thymus**

At all time points after operation, no significant difference in the product of the staining intensity and the positive rate of NF-*κ*B protein of thymus was noted between sham-operated group and model control group, between sham-operated group and treated group, as well as between model control group and treated group (*P* > .05), see Table 8.

**(5) Comparison of the Apoptosis Index of Thymus**

On 21 and 28 days after operation, the apoptosis indexes of thymus in model control group were significantly higher than those in sham-operated group (*P* < .01). At all time points after operation, no significant difference was noted between treated group and sham-operated group as well as between treated group and model control group (*P* > .05), see Table 8.

## 4. Discussion

At present, the immune organ injury in SAP and OJ has attracted more and more attention. An indispensable step to treat SAP and OJ is to help restore the function of the body's immune organs using drugs. SAP and OJ, as are diseases commonly seen in the departments of general surgery and digestive medicine, are sensitive, to a certain extent, to auxiliary treatment with Chinese traditional drugs. *Salvia miltiorrhizae* injection, an extract from Chinese traditional drug *Salvia miltiorrhizae*, possesses the vast majority of pharmacological effects of *Salvia miltiorrhizae*. Clinically, salvia miltiorrhizae injection is mainly used to promote blood flow and remove blood stasis. Some studies have proven that the tanshinol contained in *Salvia miltiorrhizae* can exert therapeutic effects on SAP and OJ through a variety of pharmacological actions [17–20]. 

When SAP develops, gut-derived endotoxins enter into the systemic circulation and cause intestinal endotoxemia which can increase the permeability of intestinal mucosa and facilitate the invasion of intestinal bacteria and endotoxins into the body. Thus, a vicious cycle is formed [29]. As a result, the activated monocytes and macrophages are further stimulated to release excessive cytokines and inflammatory mediators that can form a cascade reaction to amplify the initial signal. Ultimately, SIRS and MODS are caused [30]. Clinical studies [31, 32] prove that an insufficiency of bile salts in the intestine can cause excessive multiplication of intestinal bacteria, which can induce an increase in the production but a decrease in the activation of endotoxins. Moreover, the function of the reticuloendothelial system is inhibited. Thus, gut-derived endotoxins cannot be effectively removed and, as a consequence, enter into the blood to cause endotoxemia. The immune function injury in OJ patients is mainly contributed to endotoxin-induced damage to humoral and cellular immune responses [33]. 

We think that the lower systemic immune function in OJ patients creates conditions for the occurrence of endotoxemia which can in turn induce the damage to the body's immune function. We speculate that immune function injury results from the damage to immune organs. Comparing the pathological changes of spleen and thymus, we found that varying degrees of pathological damage were present in the spleen and thymus of SAP rats and the spleen of OJ rats in model control group but pathological damage in the thymus of OJ rats was slight. After treatment with *Salvia miltiorrhizae*, the pathological changes of spleen and thymus were mitigated, suggesting that *Salvia miltiorrhizae* is able to improve the pathological damage present in the spleen and thymus of SAP rats and the spleen of OJ rats. Additionally, the mortality rates of SAP and OJ rats in model control group were almost two times as high as those in Treated group, suggesting that *Salvia miltiorrhizae* injection can indeed reduce the mortality rate of rats. However, no statistically significant difference was observed because the sample size was too small. 

The results of this study showed that the contents of plasma endotoxin in SAP and OJ rats in model control group were significantly higher than those in sham-operated group, indicating that SAP and OJ can increase the contents of plasma endotoxin in rats. We believe that SAP and OJ can lead to an increase in the contents of endotoxin not only through causing intestinal mucosal barrier dysfunction, invasion of intestinal flora into blood, and the dissolving bacterial cell wall [34] but also through inducing hepatic injury and the decline in the phagocytic clearance function of Kupffer cells [35]. After treatment with *Salvia miltiorrhizae*, the contents of plasma endotoxin in SAP and OJ rats in treated group were significantly lower than those in model control group, indicating that *Salvia miltiorrhizae* can effectively decrease the levels of plasma endotoxin. We think that this effect may be associated with hepatic injury and intestinal mucosal barrier dysfunction in SAP or OJ, which has been proved in our previous studies (to be reported in other papers). On one hand, *Salvia miltiorrhizae* has protective effects on the liver since it can enhance the phagocytic clearance function of Kupffer cells, decrease the levels of inflammatory mediators, and scavenge free radicals. On the other hand, *Salvia miltiorrhizae* is able to protect intestinal mucosa barrier and inhibit the invasion of intestinal flora into blood. The synergistic action of these two factors can eventually antagonize the effects of endotoxin. 

Phospholipase A_2_  (PLA_2_), an enzyme to potently catalyze the hydrolysis of phospholipids, is closely related to the severity of SAP [36]. The inhibitors of PLA_2_ can improve the prognosis of SAP [37, 38]. In a certain range, the levels of PLA_2_ can sensitively reflect the extent of pancreatic injury and are therefore used as an important parameter in the diagnosis of SAP [39]. In SAP, pancreatic lysosomes release a large amount of PLA_2_. Upon activation, PLA_2_ can act on spleen and thymus mitochondria and affect the levels of phosphatidylcholine (PC) and phosphatidylethanolamine (PE), thereby altering the ultrastructure of spleen and thymus. In OJ, due to the reduction of ATP synthesis, ATP-dependent calcium pump disorders and membrane lipid peroxidation damage, cytoplasmic free calcium overload occurs. As a result, PLA_2_ is activated to promote the hydrolysis of membrane phospholipid, induce cell membrane damage, and cause the dysregulation of immune function. The results of this study showed that the contents of PLA_2_ in SAP and OJ rats in model control group were significantly higher than those in sham-operated groups. After treatment with *Salvia miltiorrhizae*, the contents of PLA_2_ in treated group were significantly lower than those in model control group, indicating that *Salvia miltiorrhizae* can effectively reduce the contents of PLA_2_ via a mechanism that may be associated with inhibiting the release of PLA_2_ from lysosomes. 

Apoptosis is a self-protective strategy employed by the body for removal of the destroyed cells through initiating programmed gene expression under certain pathophysiological conditions [39]. Bax is a soluble protein that is encoded by a recently discovered apoptosis-promoting gene. It is in the same protein family as Bcl-2 and is able to promote apoptosis [40, 41]. Our results showed that, at 12 hours after operation, the expression level of Bax protein in the spleen of SAP rats in model control group was significantly higher than that in sham-operated group, and the pathological damage in the former group was aggravated though there were no obvious changes in the apoptosis index. In contrast, at all time points after operation, the expression levels of Bax protein in the spleen and thymus of OJ rats in model control group were significantly higher than those in sham-operated group, the pathological damage was aggravated, and the apoptosis index increased. These results suggest that Bax protein is involved in the pathological and physiological processes of apoptosis present in the spleen and thymus of OJ rats. After treatment with *Salvia miltiorrhizae* injection, the expression of Bax protein in the spleen and thymus was, to varying degrees, downregulated, the apoptosis index decreased, and the pathological damage was improved in SAP and OJ rats. The therapeutic effects of *Salvia miltiorrhizae* on OJ rats were more prominent: the expression level of Bax protein (on 21 days after operation) and the apoptosis index (on 28 days after operation) of spleen in treated group were significantly lower than those in model control group. However, statistical results showed that the strength of *Salvia miltiorrhizae* was insufficient in downregulating the expression of Bax protein and inducing apoptosis in the spleen and thymus of SAP and OJ rats, and no statistically significant difference was observed. For these reasons, we think that Bax protein is not a key factor for inducing apoptosis. We surmise that cell apoptosis results from multiple contributing factors and *Salvia miltiorrhizae* only plays a weak role in modulating the expression of Bax protein and, therefore, shows no prominent effect on cell apoptosis. When SAP or OJ occurs, the apoptosis does not result from a certain factor but from multiple contributing factors. 

NF-*κ*B p65 is a protein that is able to regulate gene transcription in the nucleus and is involved in the gene regulation of multiple inflammatory factors [42]. When the body is under stress, NF-*κ*B p65 is activated and binds to specific *κ*B gene sequences, thereby promoting the gene transcription and protein synthesis of proinflammatory molecules, causing the strong expression of inflammatory cytokines such as TNF-*α* and IL-6mRNA, accelerating toxic effect on cells in multiple organs, and eventually leading to multiple organ dysfunction. In this study, we found that the staining intensity of NF-*κ*B p65 protein in the spleen and thymus of SAP and OJ rats in model control group was not significantly higher than that in sham-operated group. After treatment with *Salvia miltiorrhizae* injection, although the intensity of NF-*κ*B p65 protein in the spleen and thymus of SAP and OJ rats showed varying degrees of decline, no statistically significant difference was observed. Thus, we think that NF-*κ*B p65 is not activated or only shows weak activity in the spleen and thymus of SAP and OJ rats. Alternatively, *Salvia miltiorrhizae* may have no or show weak effect on the expression of NF-*κ*B p65 protein in the spleen and thymus of SAP and OJ rats. 

To sum up, *Salvia miltiorrhizae* injection can exert protective effects on the spleen and thymus of SAP rats and the spleen of OJ rats through reducing the contents of endotoxin and PLA_2_ in blood, improving the pathological damage present in the spleen and thymus of SAP rats and the spleen of OJ rats, and inhibiting the expression of Bax protein in the spleen of OJ rats and the apoptosis. Moreover, *Salvia miltiorrhizae* injection is also able to increase the survival rates of SAP and OJ rats. For these reasons, the application of *Salvia miltiorrhizae* for treating SAP deserves spreading and further in-depth study.

##  Note

We claimed that this paper was original and would not have any financial interest in a company or its competitor, and that all authors meet standard for authorship. We abided the ethics in this animal experimental study. The ethics committee approval of our hospital was secured for the animal study reported, and all rats have not been abused and executive mercy killing was considered when the observing time in this study was over.

## Figures and Tables

**Table 1:** Comparison of endotoxin and PLA_2 _ level of SAP groups (*M*(*Q_R_*)).

Index	Time	Sham-operated group	Model control group	Treated group
Endotoxin(EU/mL)	3 hours	0.3 (0.5)	0.5 (0.1)**	0.4 (0.1)*
	6 hours	0.3 (0.2)	0.5 (0.2)**	0.4 (0.1)^++^
	12 hours	0.3 (0.1)	0.6 (0.1)**	0.4 (0.1)*^++^
PLA_2_ (U/mL)	3 hours	31.5 (10.1)	97.0 (33.4)**	95.1 (22.9)**
	6 hours	40.4 (12.1)	117.7 (35.3)**	87.2 (24.9)**^+^
	12 hours	46.0 (12.7)	126.3 (15.7)**	87.5 (27.8)**^+^

Note: Compare to sham-operated group, *P < .01, **P < .001; compare to model control group, ^+^ *P* < .01, ^++^ *P* < .001.

**Table 2:** Pathological severity score standard of spleen.

Score	Observation indexes
0 point	Normal
1 point	Necrosis of follicle center
2 points	blood sinus dilation or arteriolar sclerosis
3 points	Necrosis of follicle center, blood sinus dilation and arteriolar sclerosis

**Table 3:** Comparision of pathological severity score of spleen and thymus in SAP group (*M*(*Q*__*R*__)).

Index	Time	Sham-operated group	Model control group	Treated group
Spleen	3 hours	0.0 (1.0)	1.0 (1.0)	0.0 (1.0)
	6 hours	0.0 (0.0)	1.0 (1.0)	0.0 (1.0)
	12 hours	0.0 (0.0)	1.0 (1.0)	0.0 (1.0)
Thymus	3 hours	0.0 (0.0)	0.0 (0.0)	0.0 (0.0)
	6 hours	0.0 (0.0)	0.0 (0.0)	0.0 (0.0)
	12 hours	0.0 (0.0)	0.0 (0.0)	0.0 (0.0)

Note: Compare to sham-operated group, *P < .01, **P < .001; compare to model control group, ^+^ *P* < .01,** ^++^ *P* < .001.

**Table 4:** Comparison of pathological indexes of spleen of SAP groups (*M*(*Q_R_*)).

Index	Time	Sham-operated group	Model control group	Treated group
Staining intensity of Bax	3 hours	0.0 (1.0)	1.0 (2.0)	1.0 (1.5)
	6 hours	0.5 (1.0)	1.0 (0.5)	1.0 (1.5)
	12 hours	0.0 (1.0)	2.0 (1.0)*	1.0 (1.0)
Product of the staining Intensity and positive rate of Bax protein	3 hours	0.0 (1.5)	2.0 (2.0)	2.0 (3.5)*
	6 hours	1.0 (3.0)	2.0 (1.5)	2.0 (3.0)
	12 hours	0.0 (2.0)	4.0 (5.0)*	2.0 (3.0)
Apoptotic indexes	3 hours	0.0 (0.0)	0.0 (0.04)	0.0 (0.01)
	6 hours	0.0 (0.0)	0.0 (0.03)	0.0 (0.0)
	12 hours	0.0 (0.0)	0.0 (0.08)	0.0 (0.0)

Note: Compare to sham-operated group,*  **P < .01,*  ***P < .001; compare to model control group, ^+^ *P* < .01, ^++^ *P* < .001.

**Table 5:** Comparison of endotoxin and PLA_2_ level of OJ groups (*M*(*Q_R_*)).

Index	Time	Sham-operated group	Model control group	Treated group
Endotoxin(EU/mL)	7 days	0.2 (0.3)	0.5 (0.1)*	0.3 (0.1)*^+^
	14 days	0.2 (0.4)	0.6 (0.1)*	0.5 (0.1)*^+^
	21 days	0.2 (0.05)	0.7 (0.1)*	0.5 (0.1)*^+^
	28 days	0.3 (0.04)	0.8 (0.1)*	0.5 (0.1)*^+^
PLA_2_(U/mL)	7 days	208.1 (26.6)	570.3 (239.6)	514.4 (104.3)
	14 days	198.5 (46.3)	653.5 (208.9)*	577.7 (129.5)*
	21 days	207.2 (38.6)	701.0 (151.7)*	593.2 (146.3)*
	28 days	203.0 (31.0)	738.6 (60.2)*	645.6 (95.1)*^+^

Note: Compare to sham-operated group,*  **P < .01,*  ***P < .001; compare to model control group, ^+^ *P* < .01,** ^++^ *P* < .001.

**Table 6:** Comparision of pathological severity score of spleen in OJ group (*M*(*Q_R_*)).

Index	Time	Sham-operated group	Model control group	Treated group
Pathological severity score of spleen	7 days	0.0 (0.0)	0.0 (0.0)	0.0 (0.0)
	14 days	0.0 (1.0)	0.0 (2.0)	0.0 (0.0)
	21 days	0.0 (0.0)	1.0 (2.0)	0.0 (0.0)
	28 days	0.0 (0.0)	0.0 (1.0)	0.0 (0.0)

Note: Compare to sham-operated group,*  **P < .01,*  ***P < .001; compare to model control group, ^+^ *P* < .01,** ^++^ *P* < .001.

**Table 7:** Comparison of pathological indexes of spleen in OJ groups (*M*(*Q_R_*)).

Index	Time	Sham-operated group	Model control group	Treated group
Staining intensity of Bax	7 days	0.0 (2.0)	2.0 (0.0)*	2.0 (1.0)*
	14 days	2.0 (2.0)	2.0 (1.0)*	2.0 (0.0)
	21 days	1.0 (2.0)	3.0 (1.0)*	2.0 (1.0)^+^
	28 days	1.0 (2.0)	1.0 (2.0)	0.0 (2.0)

Product of staining intensity and positive rate of Bax	7 days	0.0 (2.0)	4.0 (2.0)*	2.0 (3.0)*
	14 days	2.0 (4.0)	4.0 (2.0)*	4.0 (2.0)
	21 days	2.0 (4.0)	6.0 (2.0)*	4.0 (3.0)^+^
	28 days	1.0 (4.0)	0.5 (1.5)	0.0 (2.0)

Apoptotic indexes	7 days	0.0 (0.0)	0.0 (0.01)*	0.0 (0.0)
	14 days	0.0 (0.0)	0.0 (0.01)*	0.0 (0.0)
	21 days	0.0 (0.0)	0.0 (0.002)	0.0 (0.0)
	28 days	0.0 (0.0)	0.002 (0.015)*	0.0 (0.0)^+^

Staining intensity of NF-*κ*B	7 days	0.0 (0.0)	0.0 (1.0)	0.0 (0.0)
	14 days	0.0 (1.0)	0.0 (1.0)	0.0 (0.5)
	21 days	0.0 (0.0)	0.0 (0.0)	0.0 (0.0)
	28 days	0.0 (0.0)	0.0 (0.0)	0.0 (0.0)

Product of staining intensity and positive rate of NF-*κ*B	7 days	0.0 (0.0)	0.0 (1.0)	0.0 (0.0)
	14 days	0.0 (1.0)	0.0 (2.0)	0.0 (0.5)
	21 days	0.0 (0.0)	0.0 (0.0)	0.0 (0.0)
	28 days	0.0 (0.0)	0.0 (0.0)	0.0 (0.0)

Note: Compare to sham-operated group, *P < .01,*  ***P < .001; compare to model control group, ^+^ *P* < .01,** ^++^ *P* < .001.

**Table 8:** Comparison of pathological indexes of thymus in OJ groups (*M*(*Q_R_*)).

Index	Time	Sham-operated group	Model control group	Treated group
Staining intensity of Bax	7 days	0.0 (0.0)	1.0 (1.0)*	0.0 (1.0)
	14 days	0.0 (1.0)	1.0 (1.0)	0.0 (1.0)
	21 days	0.0 (0.0)	1.0 (1.0)*	0.0 (1.0)
	28 days	0.0 (0.0)	1.0 (1.0)*	0.0 (1.0)

Product of staining intensity and positive rate of Bax	7 days	0.0 (0.0)	0.0 (0.0)*	0.0 (1.0)
	14 days	0.0 (1.0)	0.0 (1.0)	0.0 (1.0)
	21 days	0.0 (0.0)	0.0 (0.0)*	0.0 (1.0)
	28 days	0.0 (0.0)	2.0 (1.5)*	0.0 (1.0)

Apoptotic indexes	7 days	0.0 (0.0)	0.0 (0.0)	0.0 (0.0)
	14 days	0.0 (0.0)	0.0 (0.01)	0.0 (0.0)
	21 days	0.0 (0.0)	0.0 (0.01)*	0.0 (0.0)
	28 days	0.0 (0.0)	0.0 (0.02)*	0.0 (0.0)

Staining intensity of NF-*κ*B	7 days	0.0 (0.0)	0.0 (2.0)	0.0 (1.0)
	14 days	0.0 (2.0)	1.0 (2.0)	0.0 (1.5)
	21 days	0.0 (1.0)	0.0 (1.0)	0.0 (0.0)
	28 days	0.0 (1.0)	0.0 (1.5)	0.0 (2.0)

Product of staining intensity and positive rate of NF-*κ*B	7 days	0.0 (0.0)	0.0 (2.0)	0.0 (2.0)
	14 days	0.0 (4.0)	2.0 (4.0)	0.0 (2.0)
	21 days	0.0 (2.0)	0.0 (2.0)	0.0 (0.0)
	28 days	0.0 (1.0)	0.0 (2.5)	0.0 (4.0)

Note: Compare to sham-operated group,*  **P < .01,*  ***P < .001; compare to model control group, ^+^ *P* < .01,** ^++^ *P* < .001.
